# Proximate and antioxidant activities of bio-preserved *ogi* flour with garlic and ginger

**DOI:** 10.12688/f1000research.17059.2

**Published:** 2019-07-12

**Authors:** Abiola F. Olaniran, Sumbo H. Abiose

**Affiliations:** 1 Department of Microbiology, Landmark University, Omuaran, Kwara, Nigeria; 2 Food Science and Technology, Obafemi Awolowo University, Ile-Ife, Osun, Nigeria

**Keywords:** Garlic, Ginger, Antioxidants, Quality, Ogi flour, Biopreservation

## Abstract

**Background**: *Ogi* from locally available cereals remains a relatively affordable complementary food in West Africa, but has a tendency to spoil due it high moisture content. This study explored effects of garlic and ginger as biopreservatives in *ogi *flour.

**Methods:** *Ogi* flour was prepared from sorghum and quality protein maize grains with different concentrations of garlic and ginger powder (2 and 4% w/w) by fermentation technique. These samples were stored for 16 weeks during which the total titratable acidity, pH, proximate composition, mineral content and total antioxidant activities were determined.

**Results**: The proximate compositions of bio-preserved *ogi* samples were relatively stable throughout storage. The addition of garlic and ginger slightly increased the ash (0.04%), crude protein and mineral contents (mg/ 100g) of the samples.  Magnesium (10.85-13.13 and 5.17-9.72); zinc (1.37-1.78 and 7.01-8.50), manganese (1.30-1.71 and 0.45-0.86) and iron (1.53-1.77 and 0.68-2.77) contents increased on addition (of garlic and ginger) to maize *ogi* and sorghum *ogi* flours respectively. The free radical scavenging activity; total phenolic and flavonoid contents increased correspondingly with the antioxidants activity.

**Conclusion:** Although not well known to *ogi* consumer, the bio-preserved ogi flours showed better nutritional values and have potential as a health food.

## Introduction

Cereal grains have become the most important plant group in terms of the human diet ^1^. Sorghum is crucial in developing countries food security because it’s one of the most important staple foods for millions of poor rural people in the semiarid tropics of Asia and Africa ^2^. Nigeria is the largest sorghum producer in 2016 in West and Central Africa region which accounts for approximately 23% of its production in Africa with forecast indicating it will become the largest sorghum grain producer in the world by 2020 ^3, 4^. Sorghum is rich in fats, protein, fiber, and minerals such as potassium, phosphorus, iron and calcium ^2^. Sorghum is gluten-free, and therefore a suitable alternative grain for people with gluten intolerance ^5^. Maize and sorghum are important cereal crops in Africa and Asia, and are consumed in various ways (porridges, snacks etc.) ^6^. They are the main staples of the majority of the Nigerian populace. Maize contains natural bioactive chemical compounds such as carotenoids and phenolic compounds ^7^. Quality protein maize (QPM) is nutritionally superior over the normal maize due to balanced amounts of essential amino acids, with a high content of lysine and tryptophan, and low content of leucine and isoleucine ^8^. Quality protein maize contains the optimal amount of amino acids in protein intake when compared with the amino acid composition to egg protein ^9^. Replacement of normal maize with quality protein maize (QPM) will impart better nutritional value to the consumers, due to its higher tryptophan (55%) and lysine (30%) content compared to normal maize. This will also contribute to food and nutrition security of the poor communities, and improve linear growth in weaning children by 19.3% where maize is consumed as staple food ^1^. *Ogi* is a thin gruel commonly used as a breakfast cereal and for infant weaning food in West Africa because it is readily available, cheap, and can be produced at household level. It can be prepared by fermentation of maize, sorghum and millet ^10, 11^. *Ogi* in paste form has the tendency to spoil because of its high moisture content ^12^. Garlic and ginger can be used biopreservative due to their antibacterial and antifungal properties to extend the shelf life of food ^13^. Garlic is found almost all over the world, and is an important herb which is now an integral part of human diet and has also been linked to health benefit such as anticancer, antioxidant, therapeutic effect, stimulation of digestion, and absorption of food ^14, 15^. Ginger is also widely used around the world in food as a spice. Both are generally regarded as safe (GRAS) for consumption in food ^16^. There is need to preserve *ogi* with naturally available spices, such as garlic and ginger, that are widely used and available. The study focused on the effect of garlic and ginger on the nutritional quality of *ogi* flour based on the proximate and mineral composition, in addition to its antioxidant activities during storage, with the aim of preserving and enhancing the nutritional level.

## Methods

### Materials

Chemicals and materials used in this study were of analytical grade namely; 1,1-diphenyl-2-picrylhydrazyl radical (Sigma Aldrich, Germany, D9132), Gallic acid (Sigma Aldrich, Germany, G7384), Quercetin (Sigma Aldrich, Germany), Folin Ciocalteau phenol reagent (Sigma Aldrich, Germany, F9252), Aluminum chloride (BDH, England,101084), potassium acetate (Sigma Aldrich, Germany, 791733-500G), sulfuric acid (38308-1EA), Ethanol (BDH, England, BDH1156-4LP), phenolphthalein (Sigma Aldrich, 74760-100ML), sodium hydroxide (Sigma Aldrich, 38227 1EA), hexane (Sigma Aldrich, Germany, C100307-2.5L) Boric acid (Sigma Aldrich, Germany,38750-1EA), Hydrochloric (Sigma Aldrich, Germany, 382801EA), Whatman No.1 filter paper (28413923) supplied by Finlab Nigeria Limited and Equilab Business solution Limited Nigeria.

### Powdered garlic and ginger preparation

Two hundred and fifty (250) grams of garlic bulbs and ginger rhizomes that were freshly harvested were washed, drained, peeled, diced into cubes, and dried at 65 °C for 12 h using hot air oven (Gallenkamp, UK). They were then ground using a grinder (Marlex Appliances PVT, Mumbai, India). Powdered garlic and ginger passed through a sieve (60 µm) (BS mesh sieves, Dual manufacturing Co. Chicago, USA) for removal of residues ^13^.

### *Ogi* preparation co-fermented with garlic and ginger

Quality protein maize (ART/98/SW06/OB/W) was obtained from the Institute of Agricultural Research and Training (I.A.R.T.), Ibadan, Nigeria, while sorghum was procured from a local market in Ile – Ife, Osun State, Nigeria. 15 kilogram of grains were examined, winnowed, and steeped separately for 3 days. Millings into smooth paste after the grains were drained and this was done with an attrition mill (No 1 Premier mill, England). Powdered garlic and ginger were added for co-fermentation to smooth paste of maize/sorghum at 2 and 4% (w/w) garlic or ginger singly and in combination (ginger-garlic), which resulted into 7 conditions. The samples were labeled A-H as follows: A: control samples (without garlic/ginger); B: *Ogi* + 2% Garlic; C: *Ogi* + 4% Garlic; D: *Ogi* + 2% Ginger; E: *Ogi* + 4% Ginger; F: *Ogi* +2% Garlic + 2% Ginger; G: *Ogi* +2% Garlic + 4% Ginger; H: *Ogi* + 4% Garlic + 2% Ginger. The slurry was evenly homogenized, then allowed to ferment spontaneously (naturally) at ambient temperature (27± 2°C) for 24 h. After fermentation the water was pressed in muslin cloth to form an *ogi* cake ^17^.

### Preparation of biopreserved *ogi* flour

*Ogi* cakes were dried for 48 h at 42± 2°C with a cabinet dryer (Gallenkamp, UK), which was then grounded into flour, cooled for 5 min at room temperature, then packaged in a pouch and sealed. The packaged samples were stored at room temperature for 16 weeks during which samples were obtained for analysis at 4 week intervals (monthly). *Ogi* flour samples were then placed on a shelf for further analysis ^18^.

### Determination of titratable acidity and pH

The total titratable acidity (TTA) of *ogi* flour was determined for all samples to quantify the acid produced during sample storage. 1g *ogi* flour was reconstituted in 10 ml of distilled water. Three drops of phenolphthalein was added as indicator; then titrated against 0.1M NaOH while gently swirling the content in the conical flask until pink colour appeared. Each ml of 0.1N NaOH used was equivalent to 90.08 mg of lactic acid. Titration readings were taken in triplicate and mean values of the readings were calculated. Total titratable acidity of lactic acid (g/ml) was calculated. A pH meter (Corning Scholar 425, UL Laboratories, Shenzhen, China) was used to determine pH values of the 5g of reconstituted flour in 50 ml of distilled water. Buffer at pH 4.0 and 7.0 were used to calibrate the pH meter. The pH of all the samples were read after stabilization of the value on the apparatus screen, the pH values were recorded in triplicate and mean values of the reading was calculated ^19^.

### Determination of proximate composition and mineral content

***Moisture content determination*.** 5 g of each sample was weighed in triplicate into pre-weighed moisture content cans. The samples were dried for 3 h at 105 °C in the Gallenkamp hot-air oven (Gallenkamp, UK) and the weight was taken. The drying continued until their weights were constant. The samples were cooled to room temperature in a desiccator and weighed. The final weight of each sample was determined ^19^. The moisture content was calculated from weight loss equation below

Moisture content = $\frac{w1-w2}{w1}\;\times \;100$ (%)

w _1_= Weight of sample before drying (g)

w _2_= Weight of sample after drying (g)

***Crude protein determination*.** 2 g of *ogi* sample was weighed into a digestion flask. Kjeltec catalyst 31835-2501AE (0.8 g) and 15 ml of concentrated sulphuric acid was added to each flask. Each flask was heated on pre heated digester set (K12, Behr LaborTechnik, Germany) at 420 °C in a fume cupboard, and digested until a clear homogenous mixture was obtained. After digestion, the flask was removed from the heater, cooled, and the content was diluted with 50 ml of distilled water. The flask was then placed in micro-kjedahl analyser (Kjelmaster K-375, Buchi, Switzerland) where it received 50 ml of NaOH automatically. The mixture was subsequently heated up to release ammonia which was distilled into a conical flask containing 25 ml of 2% (w/v) boric acid as an indicator for 4 min, the ammonia reacted with boric acid to form ammonium borate which was titrated against 0.1M hydrochloric (HCl) acid until the purplish – grey end point was attained. The percentage nitrogen content of the samples was calculated using the equation below:

Nitrogen = $\frac{A\times M\times 0.014}{weight\;of\;sample(g)}\;\times \;100$ (% g)

where A= 0.1 HCl (ml) 

Crude protein content was estimated by multiplying with the factor 6.25 (The protein content in food is estimated by multiplying the determined nitrogen content by a nitrogen-to-protein conversion factor 6.25 as the standard. AOAC, 2010). The experiment was carried out in triplicate and the means for each sample were recorded

***Crude fat content determination*.** Fat content of all the *ogi* samples was determined by a continuous extraction liquid – solid method using soxhlet extractor with a reflux condenser and a distillation flask (E914, Buchi, Switzerland). Each sample (2 g) was weighed into a fat free thimble plugged with cotton wool and placed in the appropriate chamber of the extractor. The distillation flask was filled to two third capacities with n-hexane (60–80 boiling points); the flask was boiled on a heating mantle; the distillate was collected. Thereafter, n-hexane was recovered into a clean container until almost all had been distilled. The remaining solvent in the mixture was evaporated in a Gallenkamp hot-air oven (Gallenkamp, UK) set at 70 °C. The flask was allowed to cool subsequently in a desiccator (PYREX, Corning, Inc USA after which the final weight of the flask was determined. The difference in the final and initial weight of the distillation flask represented the oil extracted from the sample ^19^.

The percentage of crude fat was obtained using the equation below:

Fat = $\frac{Final\;weight\;of\;flask-initial\;weight\;of\;flask}{weight\;of\;sample(g)}\;\times \;100$ (%)

The experiment was carried out in triplicate for each sample.

***Crude fibre determination*.** The crude fibre was determined using the weighed samples resulting from fat extraction. Each sample was transferred into conical flask and 100 ml boiling 1.25% H _2_SO _4_ added. Each beaker was heated for 30 min with periodical rotation to prevent adherence of solids to the sides of the beakers. The solution was filtered using Whatman No.1 filter paper (28413923) and rinsed with 50 ml portions boiling water; repeated trice then dried. Boiling 1.25% (w/v) NaOH solution (200 ml) was added and the mixture was boiled for 30 min after which the contents of each beaker was removed and filtered; washed with 25 ml boiling 1% sulphuric acid, three portions of 50 ml boiling water and 25 ml ethanol. The residue was dried at 100 °C to a constant weight followed by cooling in a desiccator at room temperature and weighed. The weighed residue was ignited at 600 °C in a Gallenkamp muffle furnace (Gallenkamp, UK) for 30 min, cooled in a desiccator and reweighed ^19^.

The percentage crude fibre in each sample was calculated as:

Crude fibre = $\frac{w2-w3}{w1}\;\times \;100$ (%)

W _1 _= Weight of sample (g)

W _2 _= Weight of crucible + sample (g)

W _3 _= Weight of crucible + Ash (g)

The experiment was carried out in triplicate for each sample and the average calculated for each sample.

***Total ash determination*.** The total ash (inorganic residue from the incineration of organic matter) was determined by dry ashing procedure. The samples (2 g) were weighed into a preweighed dry porcelain crucible. The samples were incinerated in a Gallenkamp muffle furnace (Gallenkamp, UK) at 550 °C for 6 h. After ashing, the remains were removed from the furnace, cooled to room temperature in a desiccator and weighed ^19^. The porcelain crucible was weighed and the % total ash weight was obtained by using the equation below:

Total ash = $\frac{weight\;of\;ash(g)}{weight\;of\;sample}\;\times \;100$ (%)

***Carbohydrate determination*.** The carbohydrate content was determined by difference. The sum of the moisture, ash, crude fiber, fat and protein of the respective samples was subtracted from 100 to obtain percentage carbohydrate ^19^. 

### Determination of mineral content

The amount of minerals present in the sample was determined as described by AOAC ^19^. The ash of the sample obtained was dissolved in 10ml of 2M HNO _3_ and boiled for 5 min, filtered through Whatman No.1 filter paper into volumetric flask. The filtrate was made up with distilled water to 50 ml and used for determination of minerals content. Zinc (Zn), Manganese (Mn), Magnesium (Mg) and Iron (Fe) were determined by using Atomic Absorption Spectrophotometer (AAS 220GF, Buck). The standard curve for each mineral was prepared from known concentrations of mineral and the mineral content of the samples was estimated from the standard curve, while sodium and potassium content were determined using Jenway flame photometer PFP7 (Cole-palmer, UK).

### Estimation of total phenolic content

Total phenolic content was determined using the modified procedure from Olaniran *et al*. ^16^. Extracts (0.1 ml) of *ogi* flour samples were pippeted into 5.9 ml distilled water; afterward, 1.0 ml Folin Ciocalteau reagent was added to 1.0 ml of the diluted extract in test tubes. The mixture was left for 5 min before addition of 2 ml of 20% (w/v) Na _2_CO _3._ After 30 min of rigorous mixing was done with a vortex mixer, absorbance was taken at 725 nm using a spectrophotometer (Model SP9, PyeUnican UK). The results were expressed as Gallic acid equivalent (GAE) using a calibration curve with Gallic acid as standard (100 mg/ ml) y = 0.0022x - 0.0292, R² = 0.9962.

### 1, 1-diphenyl-2-picrylhyrazyl (DPPH) radical scavenging activity of *ogi* flour extract

The free radical scavenging ability of *ogi* flour extracts using α, diphenyl-β-picrylhydrazyl (DPPH) were carried out following Pownall *et al.* ^20^. 1 mL of 0.3mM DPPH dissolved in ethanol in different test tubes was added to different concentrations of 1 mL of the aqueous extracts of each of the samples. The tubes were then shaken vigorously and allowed to stand for 30 min at room temperature in the dark. A control was also prepared as mention previously without the addition of the sample. Absorbance of the samples was measured at 517 nm using a UV-VIS spectrophotometer (Model SP9, PyeUnican UK) to record the changes. Free radical scavenging ability was expressed as 50% maximal radical inhibition concentration (DPPH IC _50_).

### Determination of total flavonoid content

The total flavonoid content (TFC) of *ogi* flour extract was determined following Lamien *et al*. ^21^. O *gi* flour extracts (0.5 ml) had ethanol (0.5 ml), 50 μl of aluminum chloride (10%), potassium acetate (50 μl), and water (1.4 ml) added to them, and then were incubated for 30 min at room temperature. The absorbance of the reaction mixture was read at 415 nm using spectrophotometer (Model SP9, PyeUnican UK). 0.01 g quercetin dissolved in 20 ml of ethanol was used to prepared standard quercetin solutions (y = 0.001x + 0.0018, R² = 0.992). The quantity of flavonoids present in the extracts was expressed as quercetin equivalent (QE). The quercetin solution without sample solution was used as positive control due to it’s a polyphenol content. All determinations were carried out in triplicate.

### Statistical analysis

The means were calculated and separated using MS Excel 2010 and SAS 9.4 version (2014) respectively. Means were separated with Duncan Multiple Range Test (DMRT) at 5% level of probability.

## Results and discussions

**Figure 1.**
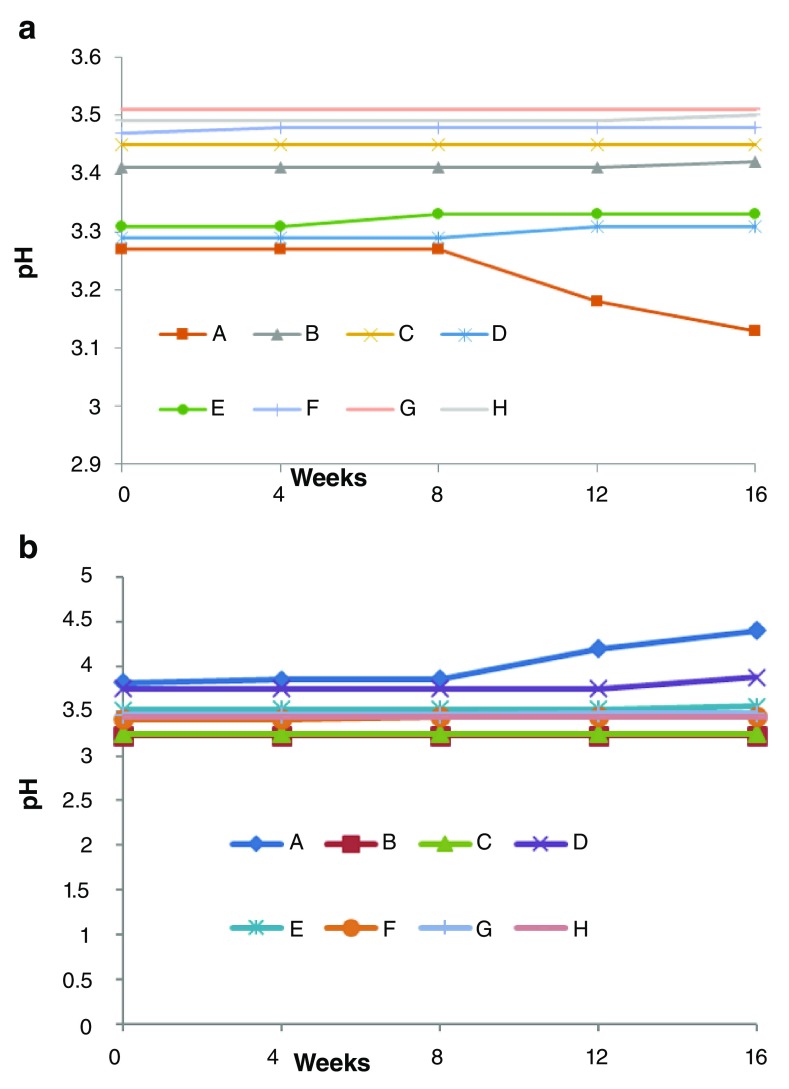
pH of *Ogi* flour made with ( **a**) maize and ( **b**) sorghum with Garlic and Ginger. Sample codes: A: *Ogi* Flour, B: *Ogi* Flour + 2% Garlic, C: *Ogi* Flour + 4% Garlic, D: *Ogi* Flour + 2% Ginger, E: *Ogi* Flour + 4% Ginger, F: *Ogi* Flour +2% Garlic + 2% Ginger, G; *Ogi* Flour+2% Garlic + 4% Ginger, H: *Ogi* Flour +4% Garlic + 2% Ginger.

**Figure 2.**
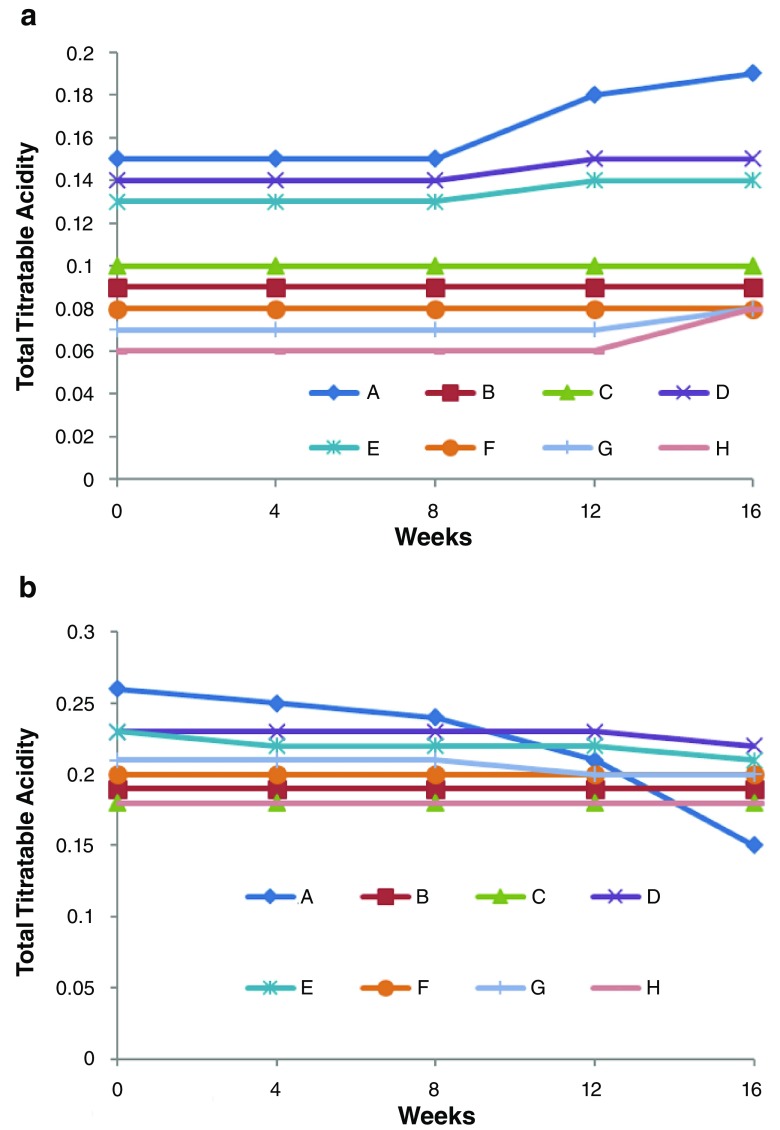
Titratable acidity *Ogi* flour made with ( **a**) maize and ( **b**) sorghum with Garlic and Ginger. Sample codes: A: *Ogi* Flour, B: *Ogi* Flour + 2% Garlic, C: *Ogi* Flour + 4% Garlic, D: *Ogi* Flour + 2% Ginger, E: *Ogi* Flour + 4% Ginger, F: *Ogi* Flour +2% Garlic + 2% Ginger, G; *Ogi* Flour+2% Garlic + 4% Ginger, H: *Ogi* Flour +4% Garlic + 2% Ginger.

**Figure 3.**
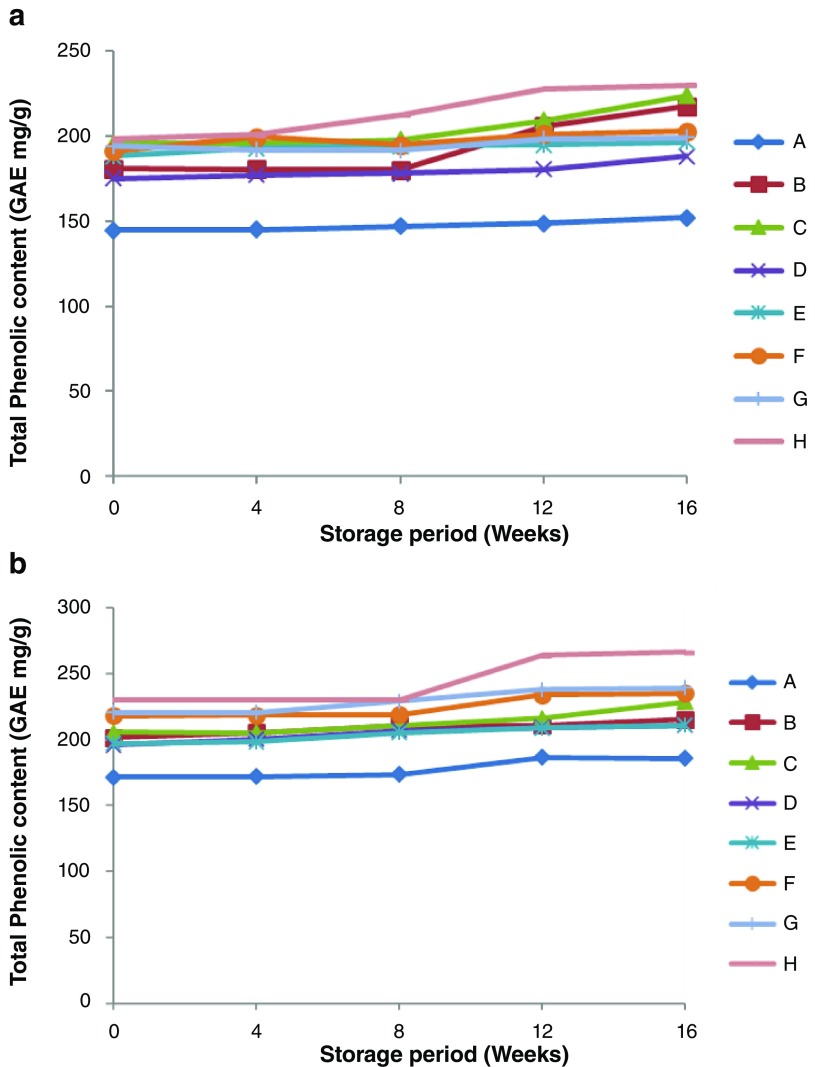
Total Phenolic Content *Ogi* flour made with ( **a**) maize and ( **b**) sorghum with Garlic and Ginger. Sample codes: A: *Ogi* Flour, B: *Ogi* Flour + 2% Garlic, C: *Ogi* Flour + 4% Garlic, D: *Ogi* Flour + 2% Ginger, E: *Ogi* Flour + 4% Ginger, F: *Ogi* Flour +2% Garlic + 2% Ginger, G; *Ogi* Flour+2% Garlic + 4% Ginger, H: *Ogi* Flour +4% Garlic + 2% Ginger.

**Figure 4.**
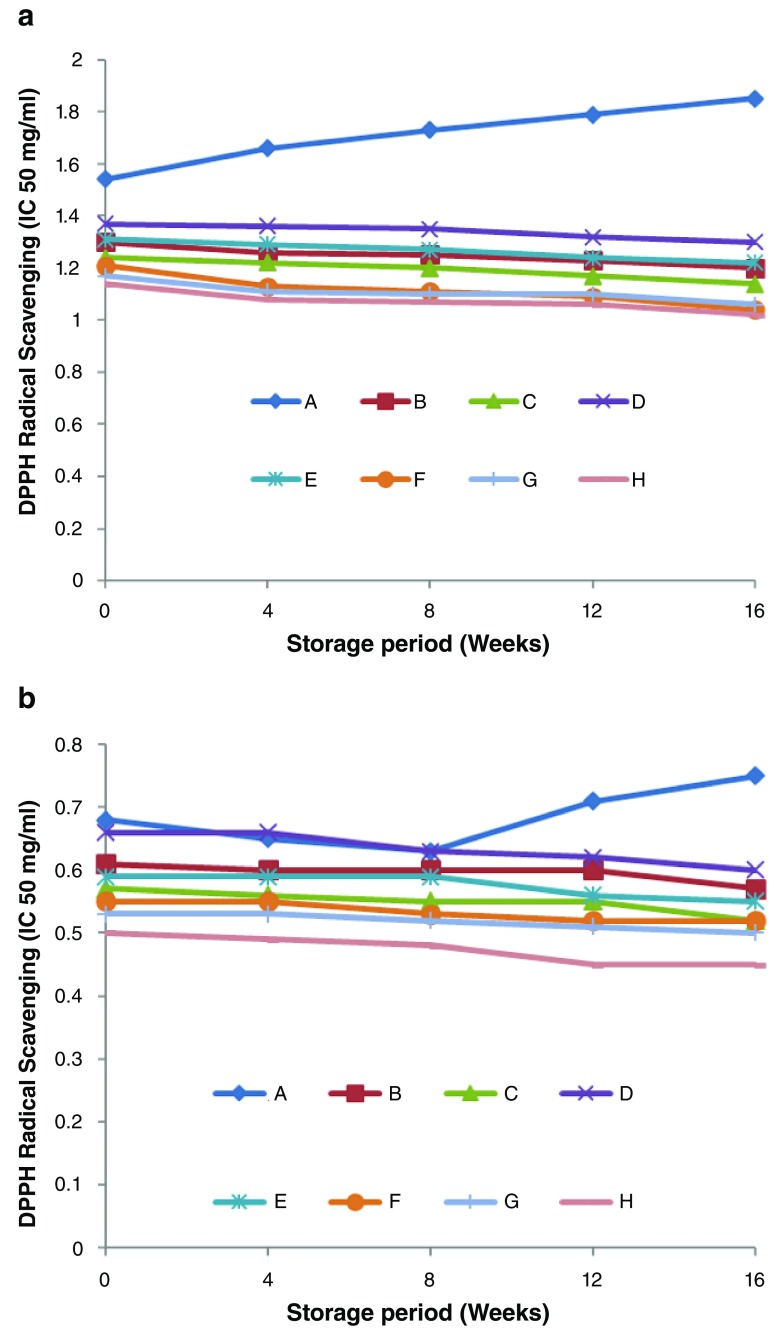
DPPH Radical Scavenging Activity *Ogi* flour made with ( **a**) maize and ( **b**) sorghum with Garlic or Ginger. Sample codes: A: *Ogi* Flour, B: *Ogi* Flour + 2% Garlic, C: *Ogi* Flour + 4% Garlic, D: *Ogi* Flour + 2% Ginger, E: *Ogi* Flour + 4% Ginger, F: *Ogi* Flour +2% Garlic + 2% Ginger, G; *Ogi* Flour+2% Garlic + 4% Ginger, H: *Ogi* Flour +4% Garlic + 2% Ginger.

**Figure 5.**
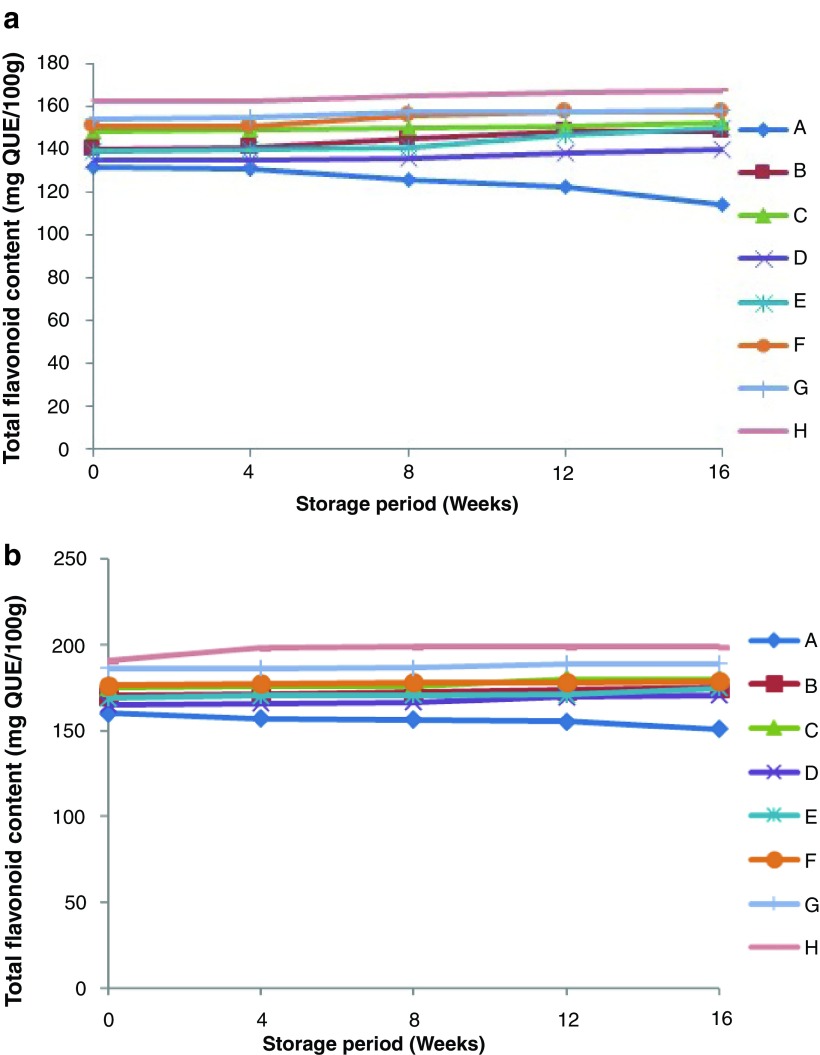
Total Flavonoid Content *Ogi* flour made with ( **a**) maize and ( **b**) sorghum with Garlic or Ginger. Sample codes: A: *Ogi* Flour, B: *Ogi* Flour + 2% Garlic, C: *Ogi* Flour + 4% Garlic, D: *Ogi* Flour + 2% Ginger, E: *Ogi* Flour + 4% Ginger, F: *Ogi* Flour +2% Garlic + 2% Ginger, G; *Ogi* Flour+2% Garlic + 4% Ginger, H: *Ogi* Flour +4% Garlic + 2% Ginger.

The total titratable acidity and pH values of all biopreserved *ogi* flour samples with only 2% garlic, 4% garlic, 4% ginger and samples with blends of 2% garlic-2% ginger, 2% garlic-4% ginger and 4% garlic-2% ginger were stable throughout the 16 weeks of storage. Addition of powdered garlic and ginger improved the stability of *ogi* flour in terms of pH and total titratable acidity (TTA) values throughout the study of 16 weeks when compared with the control. pH and total titratable acidity of the control were stable for 8 weeks during storage ( Figure 1 and Figure 2). With the exception of *ogi* flour (sorghum) containing 2% ginger, the pH was stable for 12 weeks followed by a slight increase from 3.75-3.88 till the end of storage. The addition of garlic and ginger slightly increased the ash content (0.04%), similar trends were observed in the protein content. However, in all biopreserved *ogi* samples containing garlic-ginger, a decrease in moisture content was recorded, with the lowest in *ogi* (sorghum) containing 2% garlic-4% ginger (7.70 %), when compared to the control sample (8.17 %). The moisture content of all biopreserved samples as presented in Table 1 and Table 2 ranged between 7.72-8.17%, and is less than the 10% recommendation for a floury product as reported by Ikujenlola *et al*. ^22^. The proximate composition of samples was comparable to findings reported during the production of *ogi* flour from cereal ^17, 23^. The addition also increased most of the mineral content of *ogi* samples. *Ogi* (maize) containing blends of 4% garlic and 2% ginger had the highest amount of sodium, iron, and manganese. *Ogi* (sorghum) containing blends of 4% garlic and 2% ginger has the highest amount of magnesium and zinc ( Table 3 and Table 4). The addition of garlic and ginger also enhanced the obtainable minerals in *ogi.* Garlic has been reported as rich source of minerals ^24^. The total phenolic content (TPC) of the *ogi* flour without biopreservative, increased throughout the 16 weeks of storage from 144.50, to 152.63 GAE mg/g, and 171.50-185.75 GAE mg/g, for maize and sorghum respectively. Stable TPCs for the first 8 weeks of storage were observed in biopreserved *ogi* flour (maize) samples, which then increased up to the end of the storage period ( Figure 3a and b). The total antioxidant radical scavenging activities of *ogi* flour (maize) without biopreservatives decreased throughout the period of storage from 1.54 to 1.85 mg/ml. However, the total antioxidant radical scavenging activities of *ogi* flour (sorghum) without biopreservative was stable in the first 8 weeks, and then decreased until the end of the storage period. Relatively stable total antioxidant radical scavenging activities were observed in all biopreserved *ogi* flour from maize throughout the period of storage ( Figure 4a), while in sorghum samples a gradual increase throughout the 16 weeks of storage was recorded ( Figure 4b). The total flavonoid content (TFC) of all *ogi* flour samples during the 16 weeks of storage ranged between 114.64-168.11 mg QUE/ 100g, and 150.70-198.83 mg QUE/ 100g in samples from maize and sorghum respectively. The total flavonoid content of all *ogi* flour (maize) without biopreservatives decreased throughout the period of storage from 132.01 to 114.04 mg QUE/ 100g. However, the TFC of all biopreserved samples were relatively stable throughout the 16 weeks of storage ( Figures 5a and 5b). Comparing biopreserved samples the highest total flavonoid content was observed in samples containing blends of 4% garlic-2% ginger (168.11 and 198.83 mg QUE/ 100g for maize and sorghum respectively) and the lowest in samples containing only 2% ginger (140.01 and 170.44mg QUE/ 100g for maize and sorghum respectively). Relatively stable total antioxidant radical scavenging activities were observed in all biopreserved *ogi* flour from maize throughout the period of storage. It was observed that antioxidant radical scavenging activities of all biopreserved *ogi* flour samples from sorghum gradually increased throughout the 16 weeks of storage ( Figure 5) However, the TFC of all biopreserved samples were relatively stable throughout the 16 weeks of storage. Garlic antimicrobial active such as allicin contains ajoene, methyl allyl trisulfide and diallyl disulfide which are organosulfur compounds ^25, 26^. Likewise borneol, α-pinene, linalool and camphene in ginger are responsible for its antimicrobial activities while 1, 8- cineole have been reported as key component responsible for antimycotic activity which are crucial in the preservation of food ^27^. The results of this study showed that garlic and ginger can be considered good sources of natural compounds with significant antioxidant activity. A combination of garlic and ginger exerted a synergistic effect on the radical scavenging activities of *ogi* samples with the highest effect observed in samples containing 4% garlic and 2% ginger. It also increased the total flavonoid content, and enhanced its stability in flour samples during storage.

**Table 1.** Proximate Composition of *Ogi* (maize) flour with Garlic and Ginger.

Sample/weeks	%					
	Protein	Fat	Fiber	Ash	Moisture	Carbohydrate
A _0_	10.32±0.03 ^ab^	3.41±0.01 ^a^	2.15±0.02 ^bc^	1.25±0.03 ^ab^	8.15±0.01 ^a^	74.72±0.01 ^a^
16	10.47±0.03 ^ab^	3.41±0.01 ^a^	2.18±0.02 ^b^	1.40±0.03 ^a^	8.03±0.02 ^a^	74.50±0.02 ^a^
Bo	10.45±0.01 ^ab^	3.44±0.03 ^a^	2.21±0.02 ^b^	1.33±0.02 ^ab^	8.05±0.03 ^a^	74.52±0.01 ^a^
16	10.47±0.03 ^ab^	3.41±0.01 ^a^	2.18±0.02 ^b^	1.40±0.03 ^a^	8.03±0.02 ^a^	74.50±0.02 ^a^
C _0_	10.53±0.02 ^ab^	3.52±0.01 ^a^	2.27±0.03 ^b^	1.37±0.01 ^a^	8.01±0.01 ^a^	74.30±0.01 ^a^
16	10.55±0.01 ^ab^	3.50±0.02 ^a^	2.25±0.03 ^b^	1.44±0.02 ^a^	7.98±0.01 ^a^	74.28±0.02 ^a^
D _0_	10.38±0.02 ^ab^	3.39±0.01 ^a^	2.17±0.01 ^bc^	1.27±0.03 ^ab^	8.13±0.01 ^a^	74.66±0.03 ^a^
16	10.43±0.01 ^ab^	3.42±0.03 ^a^	2.15±0.01 ^c^	1.31±0.02 ^ab^	8.15±0.01 ^a^	74.54±0.02 ^a^
E _0_	10.41±0.03 ^ab^	3.42±0.01 ^a^	2.19±0.01 ^bc^	1.30±0.02 ^ab^	8.11±0.01 ^a^	74.57±0.02 ^a^
16	10.40±0.01 ^ab^	3.41±0.01 ^a^	2.17±0.02 ^c^	1.37±0.02 ^ab^	8.14±0.03 ^a^	74.52 ±0.02 ^a^
F _0_	10.71±0.02 ^ab^	3.59±0.03 ^a^	2.33±0.01 ^b^	1.44±0.01 ^a^	7.99±0.02 ^a^	73.94±0.01 ^a^
16	10.73±0.03 ^ab^	3.58±0.01 ^a^	2.35±0.03 ^b^	1.47±0.02 ^a^	7.98±0.01 ^a^	73.91±0.03 ^a^
G _0_	10.78±0.01 ^ab^	3.65±0.02 ^a^	2.35±0.01 ^b^	1.46±0.03 ^a^	7.94±0.01 ^a^	73.82±0.02 ^a^
16	10.82±0.01 ^ab^	3.63±0.02 ^a^	2.33±0.02 ^b^	1.52±0.03 ^a^	7.91±0.01 ^a^	73.79±0.01 ^a^
H _0_	10.78±0.01 ^ab^	3.65±0.02 ^a^	2.35±0.01 ^b^	1.46±0.03 ^a^	7.94±0.01 ^a^	73.82±0.02 ^a^
16	10.93±0.02 ^ab^	3.73±0.02 ^a^	2.37±0.03 ^b^	1.58±0.02 ^a^	7.81±0.02 ^ab^	73.63±0.02 ^a^

Sample codes: A: *Ogi* Flour, B: *Ogi* Flour + 2% Garlic, C: *Ogi* Flour + 4% Garlic, D: *Ogi* Flour + 2% Ginger, E: *Ogi* Flour + 4% Ginger, F: *Ogi* Flour +2% Garlic + 2% Ginger, G; *Ogi* Flour+2% Garlic + 4% Ginger, H: *Ogi* Flour +4% Garlic + 2% Ginger.

**Table 2.** Proximate Composition of *Ogi* (sorghum) flour with Garlic and Ginger.

Sample/weeks	%					
	Protein	Fat	Fiber	Ash	Moisture	Carbohydrate
A _0_	11.03±0.01 ^a^	2.53±0.01 ^b^	3.05±0.03 ^ab^	1.20±0.01 ^ab^	8.10±0.02 ^a^	74.03±0.03 ^a^
16	11.05±0.01 ^a^	2.50±0.03 ^b^	3.11±0.02 ^ab^	1.17±0.01 ^bc^	8.17±0.03 ^a^	73.94 ±0.03 ^a ^
Bo	11.15±0.01 ^a^	2.62±0.02 ^b^	3.18±0.01 ^ab^	1.28±0.01 ^ab^	8.04±0.02 ^a^	73.73±0.02 ^a^
16	11.19±0.02 ^a^	2.58±0.03 ^b^	3.19±0.01 ^ab^	1.31±0.02 ^ab^	8.02±0.03 ^a^	73.71±0.01 ^a^
C _0_	11.18±0.01 ^a^	2.66±0.02 ^b^	3.27±0.01 ^ab^	1.30±0.02 ^ab^	8.01±0.03 ^a^	73.58±0.01 ^a^
16	11.19±0.03 ^a^	2.58±0.01 ^b^	3.19±0.02 ^ab^	1.31±0.01 ^ab^	8.02±0.02 ^a^	73.71±0.01 ^a^
D _0_	11.07±0.01 ^a^	2.55±0.03 ^b^	3.09±0.02 ^ab^	1.22±0.01 ^ab^	8.08±0.02 ^a^	73.99±0.01 ^a^
16	11.15±0.03 ^a^	2.51±0.02 ^b^	3.12±0.03 ^ab^	1.25±0.01 ^b^	8.05±0.02 ^a^	73.95±0.01 ^a^
E _0_	11.10±0.01 ^a^	2.57±0.02 ^b^	3.14±0.03 ^ab^	1.30±0.02 ^ab^	8.05±0.01 ^a^	73.84±0.01 ^a^
16	11.15±0.02 ^a^	2.55±0.02 ^b^	3.17±0.01 ^ab^	1.33±0.03 ^ab^	8.01±0.02 ^a^	73.79±0.01 ^a^
F _0_	11.21±0.01 ^a^	2.69±0.01 ^b^	3.52±0.02 ^a^	1.35±0.01 ^ab^	7.99±0.01 ^a^	73.24±0.03 ^a^
16	11.25±0.02 ^a^	2.67±0.03 ^b^	3.50±0.02 ^b^	1.38±0.01 ^ab^	7.91±0.02 ^a^	73.17±0.01 ^a^
G _0_	11.24±0.03 ^a^	2.78±0.01 ^ab^	3.60±0.02 ^a^	1.40±0.01 ^a^	7.75±0.01 ^a^	73.23±0.03 ^a^
16	11.27±0.03 ^a^	2.77±0.01 ^b^	3.57±0.01 ^a^	1.47±0.02 ^a^	7.73±0.01 ^ab^	73.19±0.03 ^a^
H _0_	11.28±0.02 ^a^	2.85±0.01 ^ab^	3.65±0.03 ^a^	1.45±0.01 ^a^	7.72±0.01 ^ab^	73.05±0.01 ^a^
16	11.30±0.03 ^a^	2.82±0.01 ^b^	3.62±0.02 ^a^	1.50±0.01 ^a^	7.70±0.03 ^ab^	73.02±0.01 ^a^

Sample codes: A: *Ogi* Flour, B: *Ogi* Flour + 2% Garlic, C: *Ogi* Flour + 4% Garlic, D: *Ogi* Flour + 2% Ginger, E: *Ogi* Flour + 4% Ginger, F: *Ogi* Flour +2% Garlic + 2% Ginger, G; *Ogi* Flour+2% Garlic + 4% Ginger, H: *Ogi* Flour +4% Garlic + 2% Ginger.

**Table 3.** Mineral Content (mg/100g) of *Ogi* (maize) flour with Garlic and Ginger.

Sample code	Sodium	Magnesium	Zinc	Iron	Manganese
A	4.69±0.01 ^d^	10.85±0.01 ^b^	1.37±0.03 ^c^	1.53±0.01 ^bc^	1.30±0.04 ^ab^
B	5.29±0.02 ^d^	11.32±0.01 ^ab^	1.48±0.01 ^c^	1.69±0.01 ^b^	1.45±0.01 ^ab^
C	5.35±0.01 ^d^	11.48±0.01 ^ab^	1.51±0.01 ^c^	1.73±0.01 ^b^	1.61±0.03 ^a^
D	5.11±0.06 ^d^	12.99±0.01 ^a^	1.40±0.02 ^c^	1.55±0.01 ^bc^	1.58±0.05 ^a^
E	5.28±0.01 ^d^	12.05±0.01 ^ab^	1.44±0.05 ^c^	1.59±0.01 ^bc^	1.60±0.05 ^a^
F	5.69±0.03 ^c^	12.58±0.01 ^ab^	1.57±0.01 ^c^	1.71±0.01 ^b^	1.65±0.03 ^a^
G	6.19±0.04 ^c^	12.61±0.01 ^ab^	1.66±0.03 ^c^	1.75±0.02 ^b^	1.69±0.02 ^a^
H	6.55±0.01 ^c^	13.13±0.01 ^a^	1.78±0.02 ^c^	1.78±0.03 ^b^	1.71±0.01 ^a^

Values are means (n = 3) ± standard deviation. Means followed by different superscripts are significantly different (p < 0.05) along column according to Duncan multiple range test. Sample codes: A: *Ogi*, B: *Ogi* + 2% Garlic, F: *Ogi* +2% Garlic + 2% Ginger, G; *Ogi* +2% Garlic+ 4% Ginger, H: *Ogi* + 4% Garlic+ 2% Ginger.

**Table 4.** Mineral Content (mg/100g) of *Ogi* (sorghum) flour with Garlic and Ginger.

Sample code	Sodium	Magnesium	Zinc	Iron	Manganese
A	12.51±0.01 ^b^	5.17±0.01 ^d^	7.01±0.01 ^b^	0.68±0.03 ^de^	0.45±0.01 ^c^
B	15.00±0.03 ^ab^	7.50±0.01 ^c^	7.18±0.01 ^b^	1.04±0.01 ^d^	0.56±0.01 ^c^
C	15.44±0.01 ^ab^	7.80±0.01 ^c^	7.64±0.01 ^ab^	1.37±0.01 ^c^	0.58±0.01 ^c^
D	15.12±0.01 ^ab^	7.10±0.01 ^c^	7.15±0.01 ^b^	0.90±0.01 ^d^	0.74±0.01 ^b^
E	15.18±0.01 ^ab^	7.40±0.01 ^c^	7.52±0.01 ^ab^	1.29±0.01 ^c^	0.77±0.01 ^b^
F	16.02±0.04 ^a^	8.05±0.01 ^c^	7.63±0.01 ^ab^	1.40±0.01 ^ab^	0.79±0.01 ^b^
G	16.13±0.01 ^a^	9.59±0.01 ^b^	7.99±0.01 ^a^	1.69±0.01 ^a^	0.88±0.01 ^b^
H	16.15±0.01 ^a^	9.72±0.01 ^b^	8.50±0.01 ^a^	1.77±0.01 ^a^	0.86±0.01 ^b^

Values are means (n = 3) ± standard deviation. Means followed by different superscripts are significantly different (p < 0.05) along column according to Duncan multiple range test. Sample codes: A: *Ogi*, B: *Ogi* + 2% Garlic, F: *Ogi* +2% Garlic + 2% Ginger, G; *Ogi* +2% Garlic+ 4% Ginger, H: *Ogi* + 4% Garlic+ 2% Ginger.

## Conclusion

This study has shown that the proximate compositions of biopreserved *ogi* flour samples were relatively stable, and that the addition of garlic and ginger slightly increased mineral content during storage. The free radical scavenging activity; total phenolic and flavonoid content also increased correspondingly with the antioxidant activity of the *ogi*. Therefore, the addition of garlic and ginger as biopreservative into *ogi* flour both singly or as blends at 2 or 4 % w/w can be used, and will not negatively affect its quality. Although not well known to *ogi* consumers, the biopreserved *ogi* flours showed better nutritional values and may be administered as a health food.

## Data availability

Underlying data is available from Figshare.

Figshare: Dataset 1. Data for Proximate and antioxidant activities of bio-preserved ogi flour with garlic and ginger. https://doi.org/10.6084/m9.figshare.7415795.v1 ^25^

License: CC0 1.0 Universal (CC0 1.0) Public Domain Dedication
