# Supplementary material for: Proximate and antioxidant activities of bio-preserved ogi flour with garlic and ginger
Source: F1000Res. 2019 Jul 12;7:1936. Originally published 2018 Dec 16. [Version 2] doi: 10.12688/f1000research.17059.2 (PMC6584973; doi:10.12688/f1000research.17059.2)
Supplement: Peer review history

# Peer review: Reviewer response for version 1

**DOI:** 10.5256/f1000research.18649.r47900

**Published:** 2019-06-17

**Authors:** Abimbola K. Arise

**Affiliations:** 1 University of Ilorin, Llorin, Nigeria

Abstract: This is well written and provides an exact overview of the work that was done.

 Introduction: This section is comprehensive and relevant. Although the references cited are relevant

 Methodology: The choice of experimental design and method are adequate and there is a clear thread between the sections.

 Results: The results are clearly presented and adequately interpreted.

 Discussion: The data analysis methods are adequate and result sufficiently discussed and compared to existing literature. This study is relevant and important particularly in the light of combating malnutrition in Africa especially among children because ogi is the major weaning food in Africa. The paper integrates well with the current research. The paper is generally well laid out and written.

 However, there is need to pay attention to tenses. Some other editorial correction have been indicated in the reviewed manuscript attached.

I confirm that I have read this submission and believe that I have an appropriate level of expertise to confirm that it is of an acceptable scientific standard.

# Peer review: Reviewer response for version 1

**DOI:** 10.5256/f1000research.18649.r45441

**Published:** 2019-03-20

**Authors:** Dolapo Oladiran

**Affiliations:** 1 Department of Consumer and Food Sciences, University of Pretoria, Hatfield, South Africa

The research aims to investigate the effect of the use of garlic and ginger as bio-preservatives on the proximate and antioxidant properties of *ogi* flour made from cereal grains (sorghum or maize). The experiment has been well designed and overall, this study contributes to the knowledge about the potential use of local spices as natural preservatives in food. Still, authors should consider the following important comments.

 There are a few typographical errors throughout the manuscript e.g. in the last paragraph under the introduction, authors should consider ‘nutritional’ instead of ‘nutritionals’ quality of *ogi*….

 Under methods specifically where ‘ *ogi* preparation co-fermented with garlic and ginger’ is described, authors should decide whether to use past tense (as in winnowed, steeped) or present continuous tense (as in draining, milling).

 The authors should consider giving further scientific depth. This can be done by expounding on the compounds present in ginger and garlic that make them good preservatives and the scientific rationale behind how these compounds exert their bio-preservative properties. If these are added, the technicality of the study especially the discussion bit would be greatly improved.

I confirm that I have read this submission and believe that I have an appropriate level of expertise to confirm that it is of an acceptable scientific standard.
